# Research on older people's health information search behavior based on risk perception in social networks—A case study in China during COVID-19

**DOI:** 10.3389/fpubh.2022.946742

**Published:** 2022-08-10

**Authors:** Chi Zhang, Wei Fang Liao, Yi Ming Ma, Chang Yong Liang

**Affiliations:** ^1^School of Public Health and Management, Wenzhou Medical University, Wenzhou, China; ^2^School of Management, Hefei University of Technology, Hefei, China; ^3^Medical Administration College, Anhui Medical University, Hefei, China

**Keywords:** health information search behavior, health risk perception, COVID-19, social network, older people

## Abstract

**Objective:**

COVID-19 has caused great loss of human life and livelihoods. The dissemination of health information in online social networks increased during the pandemic's quarantine. Older people are the most vulnerable group in sudden public health emergencies, and they have the disadvantage of infection rates and online search for health information. This study explores the relationship between the health risk perception and health information search behavior of older people in social networks, to help them make better use of the positive role of social networks in public health emergencies.

**Method:**

Based on the Risk Information Search and Processing model, and in the specific context of COVID-19, this study redefines health risk perception as a second-order construct of four first-order factors (perceived probability, perceived severity, perceived controllability, and perceived familiarity), and constructs a research model of the health risk perception and health information search behavior of older people. An online survey of people over 55 years old was conducted through convenience sampling in China from February 2020 to March 2020.

**Results:**

A total of 646 older adults completed the survey. The structural equation model showed that health risk perception is a second-order factor (H1), that health risk perception has significant positive effects on health information search behavior (H2: β = 0.470, T = 11.577, *P* < 0.001), and that health risk perception has significant positive effects on affective response (H3: β = 0.536, T = 17.356, *P* < 0.001). In addition, affective response has a significant positive mediating effect on information sufficiency (H4: β = 0.435, T = 12.231, *P* < 0.001), and information sufficiency has a significant positive mediating effect on health information search behavior (H5: β = 0.136, T = 3.081, *P* = 0.002).

**Conclusion:**

The study results indicate that the health risk perception of older people during the COVID-19 outbreak not only directly affected their health information search behavior, but also had an indirect impact on their health information search behavior by affecting affective response and information sufficiency.

## Introduction

The worldwide spread of COVID-19 has caused immense loss of human lives and livelihoods (1). During the COVID-19 pandemic, governments in many countries such as China, Italy, and the United States tried to prevent further spread by isolating confirmed and suspected cases and restricting the movement of people (2, 3). When individuals cannot obtain sufficient information from traditional approaches, they often use social networks as an alternative source of information to meet their information needs (4). During the period of forced isolation, a large amount of information related to the pandemic spread rapidly through online social networks (5).

Online social networks play an important role in disseminating health information, shaping perceptions of health risk, and providing guidance on prevention behaviors. This role was seen with the Ebola outbreak in West Africa from 2014 to 2016 and the Middle East Respiratory Syndrome (MERS) outbreak in South Korea in 2015 (6, 7). The current media era has spawned more complex information dissemination routes, a larger volume of information, and more diversified information subjects and objects (8). Misinformation or disinformation (9) may harm people's health and trigger an “information epidemic” or an “infodemic.” Information about the COVID-19 pandemic on social networks is mixed, and a large amount of unverified health information of various types has been continuously created and disseminated online during the pandemic, so that people often fail to identify scientifically valid health information (10).

The older people, as digital immigrants, are more vulnerable to information overload due to the deterioration of their physiological function, the limitations of their educational level, and their lack of network cognition (11). The World Health Organization showed that older people remain one of the most seriously affected groups during emergency situations (12) because they are not only physically vulnerable to infection, but also at a disadvantage when it comes to accessing health information. Meanwhile, the increasing health risk with age makes older people pay more attention to their health condition and health cognition level in general (13), and their desire for health information becomes more and more urgent. During the early phase of COVID-19 pandemic, it was critical for older people to obtain scientific and effective health information through social networks, which guide their lives and stabilize their mental state (14, 15).

Given this background, this study focused on 646 people over 55 years old during period of the COVID-19 outbreak in China (February 21, 2020–March 15, 2020). The study asked these questions:

(1) What was the risk perception of COVID-19 among older people during the outbreak?
(2) What was the health information search behavior of older people during the outbreak?
(3) What is the relationship between health risk perception and health information search behavior for older people?

## Theoretical background

### Online social networks

A social network is a relationship network formed by interactions between individuals or families and their relatives, friends, colleagues, neighbors, etc. (16). With the development of information technology and the emergence of social media, the online social network has become the mapping of users' interpersonal relationships in virtual space (17). These networks include those created with instant messaging software and dating software, blog or other online network platforms, media-sharing networks, and short videos (18). In recent years, diverse social networks have provided the most extensive channels for the generation, access, and sharing of health information (19).

### Health risk perception

The term “health risk perception,” which derives from “risk perception,” has not achieved a unified definition in academic circles (20). Health risk perception is an important concept composed of health and risk transmission (21, 22) and has been confirmed to have a high correlation with health behavior, which plays an important role in health behavior theory (23, 24). Some scholars have paid attention to the public risk perception when new infectious diseases occur, such as MERS or H1N1 (25, 26), and some scholars have developed a public risk perception scale for public health emergency events (27). Other investigators have identified risk perceptions as being linked to information activities (28).

Risk perception is a multidimensional concept (29). Research on the dimensions of health risk cognition is still not unified or systematic. Table 1 shows various dimensions of health risk cognition used by some scholars in recent years.

**Table 1:** Dimensions of health risk perception.

**Dimensions**	**Object of study**	**Researcher**
Severity, controllability	Radiation risk	(30)
Dread risk, risk, unknown risk	Heart disease	(24)
Likelihood, susceptibility, secondary predisposition	Cigarette smoking	(31)
Risk knowledge, personal control, environmental risk, optimistic bias	Diabetes	(32)
Perceived severity, perceived susceptibility	Sexually transmitted diseases	(33)
Possibility, severity, unpredictability, controllability	Public health emergencies	(27)
Consequences, likelihood	Influenza	(34)

In summary, in order to better understand the health risk perception of older people in the early stage of the COVID-19 outbreak, this study refers to the existing research conclusions to construct health risk perception variables from the four dimensions of perceived probability, perceived severity, perceived controllability, and perceived familiarity.

### Health information search behavior

Health information search behavior derives from information search behavior. The definition of information searching behavior that is widely recognized by scholars is, “Information searching behavior refers to the information searching activities conducted by users to meet certain target needs,” as proposed by Wilson (35). More recently, the development of user-centered online social networks has not only constructed a complex virtual interpersonal network for users, meeting their interaction and entertainment needs, but also formed a complex information base that greatly expands the health information search behaviors of users (36).

Many scholars have carried out numerous studies on the representation, content, influencing factors, search barriers, and other aspects of health information search behavior in online social networks, and drawn many scientific and effective conclusions (37). Both Manafo and Wong and Hutto et al. found that older adults do not have enough experience to construct effective online searches, that they search for information based only on their previous experience, lack the ability to evaluate the health information in social networks (38).

Therefore, we focus our research on older people and try to explore the relationship between older people's health risk perception and their health information search behavior.

### Risk information search and processing model

Griffin et al. proposed the Risk Information Search and Processing (RISP) model in 1999 based on psychology and information communication theories: the Heuristic Systematic Model and the Theory of Planned Behavior (39). The RISP model's main variables are perceived hazard characteristics, affective response, information sufficiency, information subjective norm, perceived information-gathering capacity, and relevant channel beliefs (22).

“Perceived hazard characteristics” are individuals' assessment and prediction of risk status, including perceived probability, perceived severity, institutional trust, and personal control. “Affective response” in the RISP model refers to the uncertainty, worry, and fear generated by perceived risk characteristics. “Information sufficiency” is the central variable in the RISP model, and refers to the confidence in information an individual needs to deal with a risk event [i.e., the information sufficiency threshold (39)]. It is reflected in the individual's grasp of their own risk information in the face of risks, and represents the gap between the information the individual has available and the information necessary to effectively deal with the risk. “Information subjective norm” refers to the social pressure that an individual feels when taking a specific behavior. “Perceived information-gathering capacity” measures an individual's self-efficacy in information collection. “Relevant channel beliefs” reflect the trust level of social media (22, 39).

**Figure 1:**
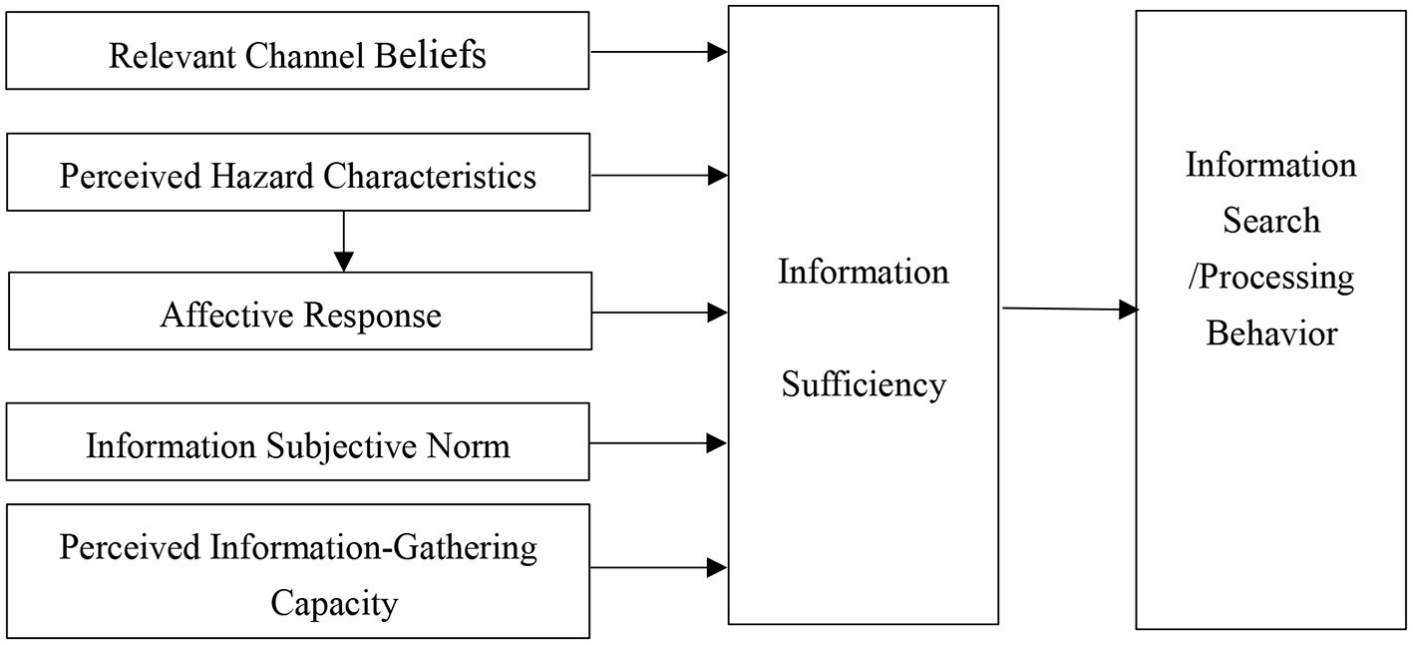
Risk information search and processing model.

The RISP model proposes that an affective response generated by perceived risk characteristics will affect the confidence one wants to have in one's knowledge about the risk (information sufficiency threshold), and that one will be motivated to have more information-seeking behavior. The specific path of the model is shown in Figure 1.

Since the RISP model was proposed, the Griffin team and other scholars have used this model to carry out a number of studies: Griffin et al. verified the impact of risk perception, worry, and subjective norms on information sufficiency in drinking water safety risk perception and fish product consumption research (22). Hunnrne et al. conducted a comparative study of information search and processing behavior for public safety risks between American and Dutch citizens and found that the RISP model has applicability and effectiveness in different cultural backgrounds (40). Hovick et al. validated cancer risk information with the RISP model (41).

Thus, the RISP model has good adaptability and should also be suitable for the study of health risk perception, providing a theoretical basis for the situational study of health information user behavior. However, some variables in the RISP model may not have a significant impact on this study and will need adjustment, as described in the following.

Considering the context of COVID-19, the study focuses on the characteristics of sudden public health events [e.g., high levels of attention to health information (15)], the impossibility of offline investigation under strict control measures (42), and the group characteristics of the older people (e.g., limited energy). Therefore, we deleted three variations: “information subjective norm,” “relevant channel beliefs,” and “perceived information-gathering capacity,” and replaced “perceived hazard characteristics” with “health risk perception.” The reasons for our changes are as follows: (1) With the RISP model, the “information subjective norm” is an important motivation to seek out and deal with non-personal risks (41). This study focuses on the risk perception of older people, which is directly related to individuals. Some studies did not consider the “information subjective norm” when they applied the RISP model to study health risk cognition, like Johnson (43). (2) Griffin et al. added media images into the RISP model and used “relevant channel beliefs” to reflect the trust level of social media (22). This study focuses on the relationship between risk perception and health information search behavior in older people facing COVID-19 and did not involve a differentiated study of various information channels. Therefore, “relevant channel beliefs” are not considered in this study. (3) Many studies have found that older people have ambiguous cognition of their own information collection ability (37). Other studies have found that self-efficacy is related to perceptions of the effectiveness of medications and confidence in self-knowledge (44), which is consistent with the “perceived familiarity” and “perceived controllability” of “health risk perception.” Therefore, “perceived information-gathering capacity” is classified as “health risk perception” and will not be studied separately. We retained the variables of “affective response” and “information sufficiency,” which will be discussed in Section Research model and hypotheses.

## Research model and hypotheses

**Figure 2:**
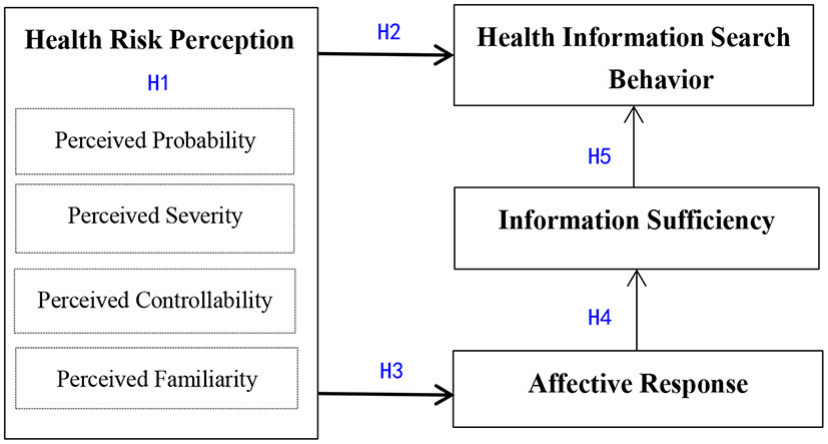
The health risk perception and health information search behavior research model.

Based on RISP theory, we propose a model of health risk perception and health information search behavior based on study of older people's online information searching behavior during the COVID-19 outbreak. Figure 2 provides a research framework for how health risk perception affects health information search behavior.

### Health risk perception and health information search behavior

Risk is commonly defined as a multiplicative combination of the probability of a hazardous event occurring and the severity of the resulting negative consequences (29). Dai et al. found that individuals have different perceptions of a risk and that their perceptions vary from one risk to another (27). Perceived severity of risk refers to an individual's assessment of the degree of harm associated with the risk (30). At the end of 2019, COVID-19 had the characteristics of being a highly unknown disease, highly contagious and having a high mortality rate. Severely ill patients were mostly older people and those with underlying diseases. Many older people began to pay attention to the risk of infection to themselves, their families, and the other people surrounding them, as well as the serious consequences after infection. Therefore, this study believes that perceived severity and perceived probability are very important parts of older people's assessment of the health risks caused by COVID-19.

In the objective result of risk, the greater the individual's ability to control the risk result, the more his risk perception will be inclined to the favorable side of the risk uncertainty result. Slovic proposed that the two dimensions of perceived possibility and risk severity (proposed in the psychometric paradigm of risk cognition) are not enough to fully reveal the characteristics of health risk cognition, and that risk controllability is also an important influencing variable (45). In their diabetes research, Walker et al. defined personal risk control as a means of behavior control taken by individuals to achieve health (32). In addition, public resources, especially medical resources provided by the government, public health funds, etc., affect the level of public health risk awareness (46). Since the outbreak of COVID-19, the Chinese government has continuously publicized a series of specific measures to prevent the spread of the virus, including wearing masks, reducing gatherings, etc. The media has continued to report on the progress of drug and vaccine research and development, and the current situation of the epidemic in various places. However, the home isolation measures taken by the government during the epidemic period made it difficult for older people to obtain information through offline information access channels, so as to assess the effectiveness of various prevention and control measures taken by the government and other public departments against the epidemic. Therefore, this study proposes that perceived risk controllability is also an important part of older people's evaluation of the health risk caused by COVID-19.

Risk familiarity is an important factor in risk assessment. Many scholars have found that risk perception is closely related to people's risk experience and knowledge of risk events (33). Slovic proposed in his risk cognition model that familiarity is an important factor affecting risk cognition, and defined familiarity as people's understanding or the visibility of risk events (45). The severity of the negative consequences that an infectious disease may cause, the possibility of contracting the infectious disease, and the ability to control its spread may all be relevant aspects of the public's assessment of the possible health risks of the infectious disease. COVID-19 was a sudden, new infectious disease. In the early days of the epidemic, the public had little knowledge of COVID-19. Limited by cognitive ability and the ability to search for information, the understanding of the infectious disease in older people was far below the average level. The familiarity of older people with COVID-19 directly affects their awareness of potential health risks.

Therefore, considering the reactions of older people during the COVID-19 outbreak as the context of this study, the first hypothesis of the study is developed:

**H1**. Health risk perception is a second-order factor of perceived probability, perceived severity, perceived controllability, and perceived familiarity.

Risk perception has been confirmed to be associated with information search behavior (47). In the health field, Patel found that risk perception has an impact on health information search behaviors in cases of breast cancer (48). With the development of social networks, Guo et al. (49) found that risk perception significantly affects the health information behavior of social media users.

The public's perception of risk has an important impact on their information searching behavior in emergency situations (7). For example, Bish and Michie found that risk perception can promote protective motivation in public health emergency events, which increases preventive behavior during infectious disease outbreaks (50). In addition, the theory of planned behavior holds that the behavioral attitude and subjective norm affect individual behavioral intention, and behavioral intention leads to behavior (51), which is consistent with the logic that health risk perception affects health information search behavior. For older people, degenerative changes in human tissues and organs inevitably bring about a weakening of the function of the immune system, and the phenomenon of “survival with disease” is more common. Older people with various chronic and underlying diseases are at higher risk of death during public health emergency events (52). As a result, older people are more concerned about their health and life safety, and they are more active in searching for health information.

Therefore, this study makes the following hypothesis:

**H2**. Health risk perception is associated with the health information search behavior of older people during public health emergency.

### Health risk perception and affective response

The estimations and judgments made by individuals in the face of the same risk event are often different (22). The RISP model divides the process of an individual facing risk information into three stages: cognition, emotion, and information search and processing (22). When individuals have differences in their perception of the possibility of risk, the severity of the consequences, the degree of trust in the risk management organization, and their own assessment of their ability to control the risk, their affective states will also be different (39). Health risk perception is the basis for the rational response of the public to public health emergency events (53). Previous studies have shown that people with different health risk perceptions differ in their negative emotions about infectious diseases (54). A positive correlation between Koreans' perception of the epidemic's severity and their negative emotions was confirmed by a study of the 2015 MERS outbreak (55).

Older people are more vulnerable to the public health crisis caused by COVID-19 because of their physical and social vulnerability (12). They tend to be less confident about their own immunity, to believe that they have a high possibility of infection and serious consequences of infection, to not be confident in epidemic control, and to not be familiar with COVID-19. Therefore, they not only have concerns about infection damaging their health, but also have concerns and fears about maintaining their personal lives and mental health. More importantly and universally, a public health crisis also weakens their social support network (12). Under these effects, older people are more likely to experience excessive worry, anxiety, and other negative emotions.

Therefore, this study makes the following hypothesis:

**H3**. Health risk perception is associated with the affective response of older people during public health emergency.

### Affective response and information sufficiency

Affective response, as a negative affective state, will affect the information sufficiency threshold, that is, the confidence one wants to have in one's information about the risk (22). This point has been verified by subsequent studies by numerous scholars. For example, Hovick et al. found that negative emotions such as anxiety mediate the relationship between health risk perception and information sufficiency, in their study on cancer risk information searching behavior (41). During the outbreak of an infectious disease, the public's negative emotions related to the disease, such as anxiety or fear, tend to be more intense, especially on social networks (56). Moreover, the panic caused by such public health emergencies can easily spread quickly, an effect that is more prominent in the information age of big data (55). For older people, their ability to process social network information is not as good as that of other age groups (57), and a large amount of health information will cause confusion over the information and health concerns (9).

On the other hand, the perception of aging among older people is more sensitive than that of other groups, and with the decline of physical function and the increase of health troubles, the concern about health is inevitably stronger than in other groups (58). During the COVID-19 outbreak, older people knew little about the newly emerged novel coronavirus and they were not confident in their own immunity. As a result, they had stronger negative emotions, such as anxiety and fear of contracting the disease and the serious consequences of infection. Negative emotions made older people more likely to have a lower understanding of health information and a greater demand for health information (i.e., higher thresholds of information sufficiency). In addition, home quarantine measures during the epidemic further limited the access of older people to information.

Therefore, this study makes the following hypothesis:

**H4**. Affective response is associated with the information sufficiency of older people during public health emergency.

### Information sufficiency and health information search behavior

The theory of RISP indicates that information sufficiency mediates the relationship between affective response and information search behavior. The threshold between the existing risk information and the information necessary to effectively respond to the risk gives individuals a strong need for information. It urges individuals to seek and process information more actively and systematically. Eventually, it affects the individual information search and processing methods (21, 22). Subsequent studies by many scholars have confirmed that individuals often meet their subjective information needs through more active information search behaviors. Public health information needs tend to increase significantly during public health emergency events, as confirmed by a survey by Tausczik et al. during the H1N1 epidemic (59). Considering the health inequalities of older adults (12), these adults show more intense health information needs and desire for understanding of health information than other groups. During the COVID-19 outbreak, traditional health information access routes (e.g., newspapers) were interrupted due to Chinese governments' home isolation measures; people had more free time and could rely more on social networks to obtain COVID-19 information. These conditions were more likely to trigger health information search behaviors on social networks.

Therefore, this study makes the following hypothesis:

**H5**. Information sufficiency is associated with the health information search behavior of older people during public health emergency.

## Research methods

In this study, a questionnaire survey was used to empirically test the health risk perception and health information search behavior research model. All items on the questionnaire were pre-validated in the existing literature and modified in combination with the specific context of the COVID-19 outbreak as well as the characteristics of the elderly population. All items included in this study were measured on a five-point Likert scale.

Before the formal survey, the validity and reliability of the questionnaire was measured in the early stages with a pre-survey sent on January 15, 2020: 276 questionnaires were distributed through Wenjuanxing (www.wjx.cn, a platform providing questionnaire distribution functions), 250 valid questionnaires were returned, and then the questionnaire was revised according to the results. This sample was only used for questionnaire corrections.

### Construct measurement

The main variables of the model in this study are health risk perception of COVID-19 (including perceived probability, perceived severity, perceived controllability, and perceived familiarity), affective response, information sufficiency, and information searching behavior related to COVID-19. The measurement indexes of the variables are shown in Table 2.

**Table 2:** The variables and questions.

**Variable**		**Code**	**Question**	**References**
Health risk perception (HRP)	Perceived probability (PP)	PP1	I may be infected by COVID-19.	(31, 54, 60)
		PP2	Someone close to me may be infected by COVID-19.	
		PP3	I think COVID-19 could happen at any time.	
	Perceived severity (PS)	PS1	I think COVID-19 is highly contagious.	(30, 60, 61)
		PS2	I think COVID-19 could kill me.	
		PS3	I don't think this COVID-19 outbreak is serious.	
	Perceived controllability (PC)	PC1	I think wearing a mask can effectively prevent infection with COVID-19.	(7, 27, 30)
		PC2	I don't think there are enough treatments for COVID-19 yet.	
		PC3	I think the spread and epidemic of COVID-19 are very difficult to control.	
	Perceived familiarity (PF)	PF1	I'm well aware of the latest developments of COVID-19.	(6, 20, 24)
		PF2	I'm well aware of the precautions against COVID-19.	
		PF3	I'm well aware of the difference between COVID-19 and a cold.	
Affective response (AR)		AR1	Your concern about COVID-19.	(22, 41, 62)
		AR2	Your fear of COVID-19.	
		AR3	Your concern about the future risks of COVID-19.	
Information sufficiency (IS)		IS1	How much do you know about COVID-19?	(22, 63)
		IS2	How much information do you need about COVID-19?	
Health information search behavior (HISB)			Please choose the option that best suits your situation according to the following statements: 1 = Very much does not match; 2 = Does not match; 3 = Average; 4 = Does match; 5 = Very much does match.	(7, 37, 38), Baidu (a search engine like Google); Baidu Index (a data sharing platform based on Baidu's massive data on netizens' behavior; www.index.baidu.com); Weibo hot word list (social media keyword search rankings).
		HISB1	I often use social networks to seek health information.	
		HISB2	During the outbreak, I was able to search on social networks for all the forms of information (text, video, pictures, etc.) I wanted about COVID-19.	
		HISB3	During the outbreak, I could search in social networks for all kinds of information I wanted about COVID-19 (source, signature, route of transmission, symptoms of infection, data on outbreaks, government measures, protective measures, research progress, supplies, people, etc.).	
		HISB4	During the outbreak, information related to COVID-19 on social networks could meet my needs.	

*Social networking platforms include but are not limited to Wechat, QQ, Nail, Momo, Tantan, Weibo, blogs, Q & A platforms (Baidu Baike, Zhihu, Chunyu Doctor, Clove Garden, etc.), forum post bars (Tianya Community, Baidu Post Bar, etc.), Douban, news clients (Toutiao, Tencent News, Sina News, etc.), and video platforms (Youku, Iqiyi, Tencent Video, Watermelon Video, Kuaishou, Douyin, etc.)*.

The questionnaire is composed of four parts. The first part gathers basic information, including age, gender, educational background, occupation, self-assessment of health status, and whether there are cases of infection in the region (city), which lays the foundation for subsequent research and analysis.

The second part is the investigation of the health risk perception of older people, which is mainly based on the studies of Slovicp et al. (20), Hayaki et al. (31), Chang (60), Dai et al. (27), Pask and Rawlins (54), Choi et al. (7), and so on. The measurement items required by this study are modified according to the actual situation. Health risk perception is divided into four dimensions in this study: possibility (individuals believe that the risk event could occur to themselves), severity (individuals perceive the severity of loss after the risk event), controllability (individuals take measures to avoid or reduce the degree of loss caused by the risk event), and familiarity (individuals' knowledge of the risk event). Each question was measured using a five-point Likert scale variable. To improve the quality of the questionnaire, PS3 and PC2 questions are set as reverse scoring questions.

The third part of the survey is about affective response and information sufficiency. Referring to the research of Griffin et al. (39), Hovick et al. (41), and Chew and Eysenbach (62), the affective response variables selected were anxiety, fear, and worry as the main affective factors; three questions measure the degree of anxiety, fear and future worry of older people about COVID-19. On a scale of 0–5, the higher the score, the higher the level of anxiety or fear or future concern. Information sufficiency uses Griffin et al. (22), Yang et al. (63), and other studies to set a total of two questions to measure the degree of understanding and demand of older adults for COVID-19 information. According to the 0–5 score, the higher the score, the higher the individual's understanding or demand for information on COVID-19. Question IS2 is set as a reverse scoring question.

The fourth part surveys the health information search behavior of older people. This part refers to the literature related to older people's health information search behavior, the Baidu index, the Weibo hot words list, the “COVID-19 Public Cognition and Information Communication Research Report” of the State Information Center and the Institute of Network Communication of Nanjing University, and the “COVID-19 Search Big Data Report” of Baidu. Health information search behavior was measured by the following four questions: “I often use social networks to seek health information,” “During the outbreak, I was able to search on social networks for all the forms of information (text, video, pictures, etc.) I wanted about COVID-19,” “During the outbreak, I could search in social networks for all kinds of information I wanted about COVID-19,” and “During the outbreak, information related to COVID-19 on social networks could meet my needs.”

### Participants

The World Health Organization defines people aged 60–74 years as young elderly, people over 75 years as middle-age elderly, and people over 90 years as long-lived elderly (64), but some studies have set people over 50 years of age as old people; for example, from a psychological development viewpoint, psychologist Anders Ericsson believes that after the age of 50, people enter psychological “old age” (65). We also take into account that China's legal retirement age for enterprise employees is 60 years for men, 50 years for women, and 55 years for female civil servants (66). Therefore, we focused on adults aged 55 years and older. In addition, during the epidemic period, China's epidemic control policies, such as “home isolation,” “one household with one person going out for shopping every 2 days,” and “concentrated isolation” for key groups (such as those returning home from high-risk areas such as Hubei Province and foreign countries), made it inconvenient to conduct offline investigations. Therefore, we used instant messaging platforms such as WeChat and QQ to conduct an online survey. In addition, older people with experience using smartphones and without literacy disorders were selected in this study to ensure that participants could fill out the questionnaire independently or with assistance.

### Data collection and procedure

In this study, the questionnaire was powered by www.wjx.cn and forwarded through WeChat and QQ. In addition, considering the physiological conditions, education, and smartphone proficiency of older people under isolation during the epidemic, the surveyor assisted participants in filling in the questionnaire, either through online assistance *via* WeChat or through the children of the respondents, working offline (i.e., the surveyor first informed the children of the respondents of the matters needing attention in filling out the questionnaire).

The formal survey, which started on 21 February 2020 and ended on 15 March 2020, collected 685 online questionnaires. In the process of data collection, 39 surveys were eliminated because some participant responses were incomplete or true, so 646 complete surveys were obtained, with an efficiency rate of 94.31%.

The participants were over 55 years old, as shown in Table 3. In addition, all participants in this study have experience searching for health information on social networks.

**Table 3:** The demographics of the sample.

	**Category**	**Number**	**%**
Sex	Male	321	49.69%
	Female	**325**	**50.31%**
Age	55–59	72	11.16%
	60–69	198	30.65%
	70–79	**223**	**34.52%**
	≥80	153	23.68%
Educational background	Never went to school	89	13.78%
	Elementary school	113	17.49%
	Middle school	141	21.83%
	High school	**154**	**23.84%**
	College	133	20.59%
	Master's degree	16	2.48%
Professional	Civil servants	52	8.05%
	Public institution personnel	98	15.17%
	Employees of enterprises	126	19.50%
	Farmers (migrant workers)	**161**	**24.92%**
	Self-employed or private owners	62	9.60%
	Housewives	90	13.93%
	Soldiers	16	2.48%
	Retired/unemployed	41	6.35%
Health	Good	294	45.51%
	Not bad	**305**	**47.21%**
	Bad	47	7.28%
Infection in region	Yes	**347**	**53.71%**
	No	225	34.83%
	I don't know	74	11.46%

*“Infection in region” refers to the judgment of respondents on whether there were cases of infection in their area (city or township). The bold values indicate the maximum value of the item*.

## Results

### Measurement model

First, we measured how well the model fits. The results are shown in Table 4. As shown in Table 4, the result of SRMR is <0.8, and the values of d_ULS and d_G are both less than the corresponding value of 95% of the bootstrap quantile (HI95). Therefore, confirmatory composite analysis results show that the model has a good fit (67). The maximum variance expansion coefficient (VIF) was 5.440, much lower than the prescriptive diagnosis of 10.0 (67). This result shows that there is no multicollinearity problem in this model.

**Table 4:** Confirmatory composite analysis results.

**Discrepancy**	**Value**	**HI95**	**Conclusion**
SRMR	0.046	0.098	Supported
d_ULS	0.481	2.234	Supported
d_G	0.411	0.493	Supported

*HI95, 95% of bootstrap quantile*.

In this study, we used Harman's one-factor test to test the possible common method bias (67). The test results show that the largest factor accounts for 50.451, which is acceptable according to the recommendations of Fuller et al. (68). Therefore, the common method bias problem in this study is very small.

The sample data was analyzed by descriptive statistics, and the results are shown in Table 5. According to the analysis results, the average values of all variables in the model range from 3.506 to 3.987, and the standard deviation ranges from 1.089 to 1.330. In addition, the Cronbach's α of each latent variable is higher than the threshold value of 0.7, which indicates that the data reliability of each latent variable is high.

**Table 5:** Reliability, validity, and factor loading.

**Latent variable**		**Item**	**Mean**	**SD**	**Loading**	**Alpha**	**CR**	**AVE**
Health risk perception (HRP)	Perceived probability (PP)	PS1	3.653	1.250	0.910	0.859	0.914	0.780
		PS2	3.684	1.303	0.945			
		PS3	3.690	1.259	0.921			
	Perceived severity (PS)	PC1	3.617	1.168	0.853	0.868	0.919	0.792
		PC2	3.532	1.089	0.939			
		PC3	3.580	1.145	0.876			
	Perceived controllability (PC)	PP1	3.609	1.256	0.878	0.916	0.947	0.856
		PP2	3.551	1.174	0.896			
		PP3	3.659	1.141	0.875			
	Perceived familiarity (PF)	PF1	3.814	1.194	0.873	0.888	0.930	0.817
		PF2	3.688	1.283	0.938			
		PF3	3.569	1.297	0.900			
Affective response (AR)		AR1	3.945	1.310	0.917	0.912	0.945	0.851
		AR2	3.987	1.286	0.940			
		AR3	3.928	1.330	0.911			
Information sufficiency (IS)		IS1	3.648	1.186	0.893	0.783	0.902	0.821
		IS2	3.506	1.109	0.919			
Health information search behavior (HISB)		HISB1	3.608	1.210	0.821	0.897	0.929	0.767
		HISB4	3.625	1.258	0.891			
		HISB5	3.744	1.300	0.946			
		HISB6	3.702	1.238	0.840			

The validity of the questionnaire can be divided into convergent validity and discriminant validity (69). Convergent validity refers to the similarity of measurement results when different measurement methods are used to measure the same characteristics (70). Factor load, combined reliability (CR), and average variance extracted (AVE) are three effective indexes to test the convergent validity. The factor load of the observed variables exceeds the recommended value of 0.6, which indicates that the observed variables of the model are highly correlated with the structural variables (71). The data shows that the factor load of each observation variable exceeds the recommended value of 0.6. The CR describes the degree to which the observed variable determines the latent structure, and a value >0.7 can be considered as having a good internal consistency of the variable (71). As shown in Table 5, all CR values are >0.7. The AVE reflects the total difference of potential structure, and a value >0.5 indicates that the observed variables in this model explain each measurement dimension well. The AVE value in this study is >0.5 (72).

Discriminant validity is the extent to which a construct is truly distinct from other constructs by empirical standards. There are three main ways to evaluate discriminant validity: cross-loadings, the Fornell-Larcker criterion, and the heterotrait-monotrait (HTMT) ratio; *A Primer on Partial Least Squares Structural Equation Modeling (PLS-SEM)* (2nd edition) was used (73). Discriminant validity can be measured by the square root of each AVE observation variable. The square root of AVE observation variables is greater than the correlation coefficient between AVE observation variables and other observation variables, which indicates that each observation variable has a higher discrimination degree (74). In this study, the square root of AVE observation variables is greater than the correlation coefficient between AVE observation variables and other observation variables. Table 6 shows the details. In summary, the questionnaire has good reliability and validity.

**Table 6:** Fornell-Larcker criterion.

	**AR**	**HISB**	**IS**	**PC**	**PF**	**PP**	**PS**
AR	0.922						
HISB	0.358	0.876					
IS	0.435	0.432	0.906				
PC	0.398	0.440	0.508	0.890			
PF	0.427	0.448	0.472	0.509	0.904		
PP	0.453	0.415	0.494	0.477	0.529	0.883	
PS	0.398	0.433	0.498	0.492	0.423	0.460	0.925

### Structural model and discussion

#### Total effect analysis

**Figure 3:**
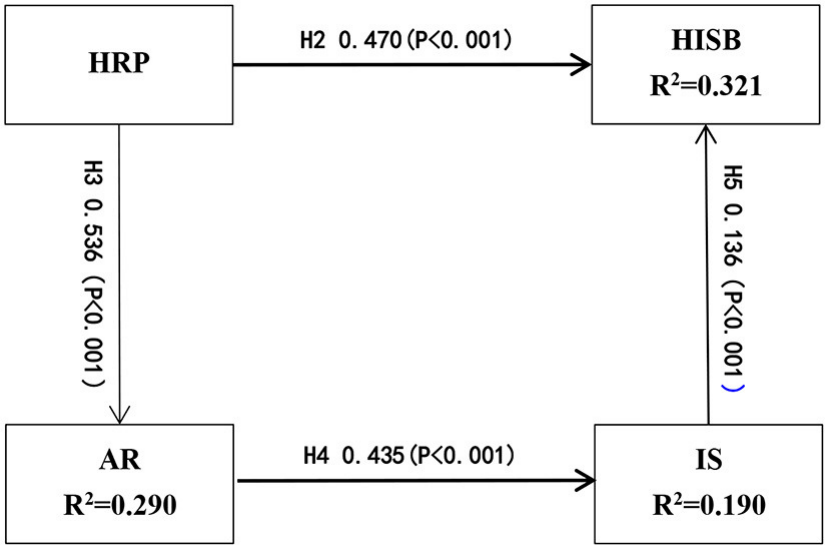
Model results.

We calculate the model path coefficients using SmartPLS 3.3.2. The results are shown in Figure 3 and Table 7.

**Table 7:** The results of hypothesis testing.

**Hypothetical path**	**β**	**T value**	***P* value**	**Conclusion**
H2: HRP -> HISB	0.470	11.577	<0.001	Support
H3: HRP -> AR	0.536	17.356	<0.001	Support
H4: AR -> IS	0.435	12.231	<0.001	Support
H5: IS -> HISB	0.136	3.081	0.002	Support

*β is used to generate the best component score of the predictive effectiveness of the potential independent variable against the related potential dependent variable or the observed variable (75)*.

The results show that H2, H3, H4, and H5 are supported. The model prediction ability of this study is evaluated by the internal model interpretation utility R^2^. The greater the value of R^2^, the stronger the explanatory ability of the measured variable to the latent variable. In this study, 32.1% of the model is explained by health information search behavior, 29.0% by affective response, and 19.0% by information sufficiency.

In addition, we use blindfolding to test the predictive correlation of the model. Q^2^ is used to analyze the validity of structural models; when the Q^2^ value is greater than zero, the model has the ability to predict, and the greater the Q^2^ value, the stronger the correlation of the model prediction (74). In this study, all of the Q^2^ values (AR = 0.104, IS = 0.141, and HISB = 0.280) are greater than zero. In summary, the model has a good correlation of prediction.

#### Mediating effect analysis

We used the Bootstrapping function of SmartPLS 3.3.2 to verify the mediating effects of affective response on health risk perception and information sufficiency, and information sufficiency on affective response and health information search behavior (76). The sample size of bootstrapping is set at 5,000. The results of the mediating effect analysis showed that the total effect, direct effect, and indirect effect are significant (*P* < 0.001). The specific results are in Table 8.

**Table 8:** The mediating effects of affective response and information sufficiency.

**Path**	**β**	**T value**	**VAF**	**Conclusion**
HRP -> AR -> IS	0.233	T = 8.085	33.86%	Partial mediation
AR -> IS -> HISB	0.148	T = 6.368	70.47%	Partial mediation

According to a standard test of the mediating effect proposed by Hair et al. (77), the ratio of indirect effects to total effects (VAF) can be used to measure the strength of the mediating effect. It is generally considered that the ratio of indirect effects to total effects is >80% for complete mediation, 20–80% for partial mediation, and <20% for no mediation. The results show that the mediating effects of affective response between health risk perception and information sufficiency account for 33.86%, and the mediating effects of information sufficiency between affective response and health information search behavior account for 70.47%, both >20%. Therefore, affective response is partially mediated between health risk perception and information sufficiency, and information sufficiency is partially mediated between affective response and health information search behavior.

#### Second-order analysis

In this study, health risk perception is a second-order factor divided into perceived probability, perceived severity, perceived controllability, and perceived familiarity. For hierarchical latent variable models, two approaches are commonly adopted to estimate the parameters by using PLS-SEM: the repeated indicator and two-stage approaches (78). In this study, the two-stage approach was used because the first-order model had four formative constructs. We estimated a repeated indicator model in the first stage and used the first-order construct scores in a separate second stage (79). The results of the second-order analysis are illustrated in Table 9. HRP is a second-order variable composed of four first-order potential structures: PC, PF, PP, and PS, supporting H1.

**Table 9:** Weights of first-order constructs on information quality second-order construct (examining H1).

**Hypothetical path**	**β**	**T value**	***P* value**	**Conclusion**
PC -> AR	0.121	2.600	0.009	Support
PC -> HISB	0.144	3.127	0.002	Support
PF -> AR	0.177	3.634	<0.001	Support
PF -> HISB	0.184	3.796	<0.001	Support
PP -> AR	0.229	4.666	<0.001	Support
PP -> HISB	0.105	2.315	0.021	Support
PS -> AR	0.158	3.137	0.002	Support
PS -> HISB	0.169	3.539	<0.001	Support
AR -> IS	0.435	12.231	<0.001	Support
IS -> HISB	0.136	3.081	0.002	Support

## Discussion

### Key findings

Overall, the data analysis results show that all the hypotheses in this study are valid. The results provide the following key findings:

First, older people's health risk perception for COVID-19 is a second-order construct composed of four first-order (i.e., lower-order) constructs, including perceived probability, perceived severity, perceived controllability, and perceived familiarity. These four first-order constructs can well represent the impact of health risk perception on health information search behavior and affective response.

Second, during the early phase of COVID-19 pandemic, the health risk perception of older people not only directly affected their health information search behavior, but also influenced their health information search behavior indirectly by affecting their affective response and information sufficiency. The higher the individual's health risk perception level, the stronger the affective response, the lower the individual's grasp of their own health information, and the more health information needed (the higher sufficiency threshold); and thus, the more active the health information search behavior.

Third, affective response plays an intermediary role in health risk perception and information sufficiency, and information sufficiency plays an intermediary role in affective response and health information search behavior.

Specifically, we found that health risk perception had significant positive effects on health information search behavior. The high infectivity and mortality rates of COVID-19, as well as the rapid spread of epidemic information through social networks, greatly affect the risk perception of COVID-19 among older people. The older people, who perceive themselves to be at higher risk of infection and severe consequences, which is coupled with their lack of confidence in epidemiological control and knowledge of COVID-19 information, are more motivated to seek health information.

We also found that affective response and information sufficiency have a mediating effect. The older people have declining physiological functions, low immunity, and many underlying diseases, which makes them more likely to be infected with infectious diseases and makes the consequences of infection more serious. During the COVID-19 outbreak, the new coronavirus pneumonia was a new infectious disease with a high infection rate and high mortality rate, and older people had insufficient awareness. The increase in reported cases of infection and the spread of unproven health information have placed older adults in an information disadvantage, making them more difficult to recognize health risks. Older people often show a more sensitive affective response to public health emergencies. They are more likely to experience negative emotions such as anxiety, fear, and future worry, which are manifested by a lower understanding of health information, stronger demand for health information, and higher information sufficiency threshold. Therefore, when the sufficiency threshold is higher than the health information the older person feels he or she currently has, more active health information search behaviors will be motivated. In addition, due to the influence of the Chinese governments' home isolation measures, traditional access to health information (such as newspapers) was interrupted, but older people had more free time and could rely more on social networks to obtain information on the novel coronavirus, a state that was more likely to trigger their social network health information search behavior.

### Implications

COVID-19 is a new, intense, highly contagious, and high mortality infectious disease (80). In this study, we established a research framework for the correlation between health risk perception and health information search behavior in the context of COVID-19, which aims to provide a reference for future research on the correlation between public health risk perception and health information search behavior in the case of major infectious diseases. There are two main contributions: theoretical and practical.

The theoretical contributions of this study have three aspects. First, considering the characteristics of the early outbreak period of COVID-19 and the characteristics of older people's perception of public health emergencies, we set the health risk perception variable as a second-order construct, including four first-order constructs: perceived risk probability, perceived risk severity, perceived risk controllability, and perceived risk familiarity; We formed measurement items with reasonable reliability and validity. This measurement method of health risk perception improves on the measurement methods of health risk in previous studies on chronic diseases, bad living habits, conventional infectious diseases, etc. (31–33), and provides an effective reference for the development of public risk perception measurement tools in public health emergencies.

Second, we introduced health risk perception into the study of health information search behavior, replaced the perceived hazard characteristics in the RISP model with health risk perception, and focused on the internal correlation between older people's individual health risk perception and health information search behavior during the early phase of COVID-19 pandemic. Our results show that although older people are a vulnerable group in the information age (81), they will have active online health information search behaviors in the face of health risks, and their health risk perception not only directly affects their health information search behavior, but also indirectly affects their health information search behavior through affective response and information sufficiency. This meaningful discovery is not only a verification of the adaptability of the RISP model, but also a modification about the specific contextual application of the RISP model in the context of the global pandemic. More importantly, it provides a reference research framework for interactive research on older people's social network health risk perception and health information search behavior in specific epidemic situations from the three levels of cognition, emotion, and behavior, and expands the depth of RISP model research.

Finally, we found the mediating role of affective response and information sufficiency. During the COVID-19 outbreak, older people who are vulnerable to health risks were more likely to experience anxiety, fear, worry, etc.; lower understanding of health information; and greater demand for health information (higher thresholds of information sufficiency). In addition, home quarantine measures during the epidemic further limited the access of older people to information. When the information adequacy threshold is higher than the health information currently possessed by older people, they will be more motivated to search for health information. This mediation path fully reflects the uniqueness of older people's health information search behavior on social networks during the epidemic. The results are helpful for us to further understand the psychological and informational behavior characteristics of older people in sudden public health events.

In addition to the theoretical significance, this study also has practical significance, as detailed below.

First of all, our research helps older people take the initiative in public health emergencies. Older people can obtain scientific health information on social networks to protect their own health and respond to an outbreak. Older people should be encouraged to improve their ability to search online and screen health information.

Second, health information providers can provide personalized and accurate health information services for older people based on health risk perception. For example, for low-risk awareness users, providers can push information on health risk hazards, epidemic conditions, and other related information to raise users' awareness; for high-risk awareness users, providers can provide relevant health information or facts about effective treatment to relieve users' tension. In addition, information sufficiency has a significant effect on health information search behavior, which suggests that information service providers should focus on improving this information sufficiency and attach importance to the health information needs of older people, as well as realizing the great potential. For example, governments and service providers should build a “suitable for older people” health information network platform. The platform should be designed to reduce the complexity of searching, with features such as enlarged font, automatic voice broadcasting, short video explanations, and voice commands for entering searches.

In addition, it is very important for public health departments to control the quality of the mass of information provided by social media. During public health emergencies, massive amounts of information are spread on social media (9), but the quality of the information is worrisome (82). In particular, older people whose health information recognition and processing ability is weak need more information assistance from the government. For example, authorities could detect false epidemic information and flag it as problematic, conduct quality certifications of health websites, improve the reporting channels of health information on the Internet, and disclose accurate epidemic information while exposing false health information. In addition, the government could encourage authoritative health websites and popular science platforms to create separate sections or versions for older people.

### Limitations and further studies

In this study, which was affected by the early phase of the COVID-19 pandemic, an online network questionnaire was used instead of a traditional experimental study of search behavior; and the search behavior described, using a self-rating scale, was not as accurate, objective and sufficient as traditional experimental data would have been. In addition, the Chinese government implemented a strict home quarantine policy from January to April 2020 (42), and an offline survey was not possible in this quarantine period. Therefore, non-random sampling was used in this study, and the survey subjects were limited to older people who used smartphones and had no reading or writing impairments. The study ignored older adults who were less educated, did not use smartphones or were in poor health. Moreover, the effective sample size was only 646 cases; such a small sample size is not conducive to the development of the study results. Therefore, future research will expand the scope of the questionnaire and improve the sample representation.

There may be questionnaire comprehension bias in the elderly group due to their decline in physiological function, weakened comprehension and education level limitations. In future research, experimental research will be considered to determine the characteristics of the health information search behavior of older people. Further research can also explore whether the health risk cognition of older people changes before and after the search, and the influencing factors of the change.

Furthermore, the study did not take into account the influence of different social media platforms (including instant messaging software, dating software, short video platforms, and so on) on older people's information searching behavior, which can be further analyzed and discussed in subsequent studies. In addition, from January to March 2020, the cumulative confirmed cases and cumulative deaths of COVID-19 varied greatly in different regions of China. The Hubei province had the highest number of confirmed cases and cumulative deaths, followed by Zhejiang, Guangdong, Henan, and Hunan (83). However, this study did not fully consider the influence of regional infection rates on the information search behavior of older people. This is also a point that further research should focus on.

In addition, this study lacked a comparative study and analysis between older people and young people and did not consider the effects of aging characteristics on health risk perception and health information search behavior. In future research, we can conduct a differential study on the impact of health risk perception on online health information search behavior among different age groups, and add research variables of aging characteristics into the study, such as vision, hearing, thinking ability, and chronic diseases.

Finally, our study focused on the association between health risk perception and health information search behavior in older adults during the COVID-19 outbreak. Although COVID-19 is a global public health emergency, its specificity limits the results of our study. Due to the diversity of public health emergencies, the impact of a pandemic may be different from the impact of other emergencies. We cannot also compare COVID-19 with another pandemic. In future studies, we can look for more data to study the association between health risk perception and health information search behavior of older people in a variety of public health emergencies. This will make a huge contribution to the health of all people.

## Conclusion

Considering the vulnerability of older people to public health emergencies, especially in the context of COVID-19, we proposed a research framework for health risk perception and health information search behavior based on the RISP model. Using a questionnaire and verifying model, we found that the health risk perception of older people during the COVID-19 outbreak not only directly affected their health information search behavior, but also had an indirect impact on their health information search behavior by affecting affective response and information sufficiency: the stronger the affective response, the lower the grasp of health information and the more health information needed (the higher sufficiency threshold), and thus the more active the health information search behavior. This study also redefined the variable of health risk perception and provided a tool for the measuring health risk perception during the epidemic. It provides valuable advice to older people, government public health departments, and health information service providers—advice that can improve the active role of online social networks during sudden public health events.

## Data availability statement

The raw data supporting the conclusions of this article will be made available by the authors, without undue reservation.

## Ethics statement

The studies involving human participants were reviewed and approved by Research Ethics Committee of Wenzhou Medical University. The patients/participants provided their written informed consent to participate in this study.

## Author contributions

CZ, WL, and YM contributed to conception and design of the study. CZ and WL organized the database and wrote the first draft of the manuscript. WL performed the statistical analysis. YM wrote sections of the manuscript. All authors contributed to manuscript revision, read, and approved the submitted version.

## Funding

This work was supported in part by Wenzhou Key Research Base of Social Sciences (18jd07) and the National Natural Science Foundation of China (71771075).

## Conflict of interest

The authors declare that the research was conducted in the absence of any commercial or financial relationships that could be construed as a potential conflict of interest.

## Publisher's note

All claims expressed in this article are solely those of the authors and do not necessarily represent those of their affiliated organizations, or those of the publisher, the editors and the reviewers. Any product that may be evaluated in this article, or claim that may be made by its manufacturer, is not guaranteed or endorsed by the publisher.
